# Effect of Extraction Procedures with Ultrasound and Cellulolytic Enzymes on the Structural and Functional Properties of *Citrus grandis* Osbeck Seed Mucilage

**DOI:** 10.3390/molecules27030612

**Published:** 2022-01-18

**Authors:** Yu-Cheng Yeh, Lih-Shiuh Lai

**Affiliations:** Department of Food Science and Biotechnology, National Chung Hsing University, 145 Xingda Road, Taichung 40227, Taiwan; neverever20140101@gmail.com

**Keywords:** seed mucilage, enzyme-assisted extraction (EAE), ultrasound-assisted extraction (UAE), ultrasonic-assisted enzymatic extraction (UAEE)

## Abstract

The structural and functional properties of *Citrus grandis* Osbeck (CGO) seed mucilage by different extraction practices, including conventional citrate buffer, ultrasonic-assisted (UAE), enzymatic-assisted extraction (EAE) with cellulase or Celluclast^®^ 1.5 L and various ultrasonic-assisted enzymatic extraction (UAEE) procedures were investigated. It was found that CGO seed from agricultural and processing byproducts is an excellent new source of high methoxyl pectin with quite high intrinsic viscosity (about 108.64 dL/g) and molecular weight (about 1.9 × 10^6^) as compared with other pectin sources. UAEE with Celluclast^®^ 1.5 L enhanced the extraction yield most pronouncedly (about 2.3 times). Moreover, the monosaccharide composition of CGO seed mucilage is least affected by EAE with Celluclast^®^ 1.5 L. In contrast, EAE with cellulase dramatically reduces the galacturonic acid (GalA) content to less than 60 molar%, and increases the glucose (Glc) content pronouncedly (to about 40 molar%), which may be considered as an adverse effect in terms of pectin purity. Though extraction procedures involved with ultrasound and cellulolytic enzymes generally show a decrease in GalA contents, weight average molar mass and intrinsic viscosity, EAE with Celluclast^®^ 1.5 L is least affected, followed by UAE and UAEE with Celluclast^®^ 1.5 L. These features can be leveraged in favor of diversified applications.

## 1. Introduction

Polysaccharide gums and mucilage are widely used for various food, pharmaceutical and cosmetic systems. Their structural and physicochemical characteristics are linked to the biological origins and extraction or modification practices. The growing demand for sustainable food ingredients also leads to researches on understanding the performance of polysaccharides and mucilage from nonconventional sources and wastes of industrial processing, which provides new opportunities for their ecofriendly usage [1,2].

Seed coat is commonly composed of the non-adherent and adherent mucilage, and the extraction and isolation efficiency may be promoted through technologies such as enzyme-assisted extraction (EAE) [3,4,5,6,7], microwave-assisted extraction (MAE), and ultrasonic-assisted extraction (UAE) [8,9,10,11,12,13,14,15]. Furthermore, as compared with single extraction, combination of different extraction technologies, such as ultrasonic-assisted enzymatic extraction (UAEE), may show synergistic effect [16,17,18,19]. The seed of *Citrus grandis* Osbeck (CGO) contains significant amounts of mucilaginous substances. However, studies about the physicochemical properties of this potential mucilage are quite limited. Wang et al. [20,21] isolated a low-degree (11.94%) esterified homogalacturonan from the outer-layer seed hull of CGO by water extraction and found that it exhibited some bioactivities such as antioxidant activity and stimulation of the proliferation of NIH3T3 cells. The objective of this study is to further investigate the structural and functional properties of the mucilage from the seed of CGO as a function of extraction procedures involving ultrasonic and cellulolytic enzyme-assisted extractions (cellulase or Celluclast^®^ 1.5 L). It is expected that understanding the impact of UAE, EAE and UAEE on the structural and functional properties of such a nonconventional hydrocolloid like CGO seed mucilage may promote a diversified scheme for future sustainability due to their green and safe nature.

## 2. Results and Discussion

### 2.1. Extraction Yield

**Figure 1:**
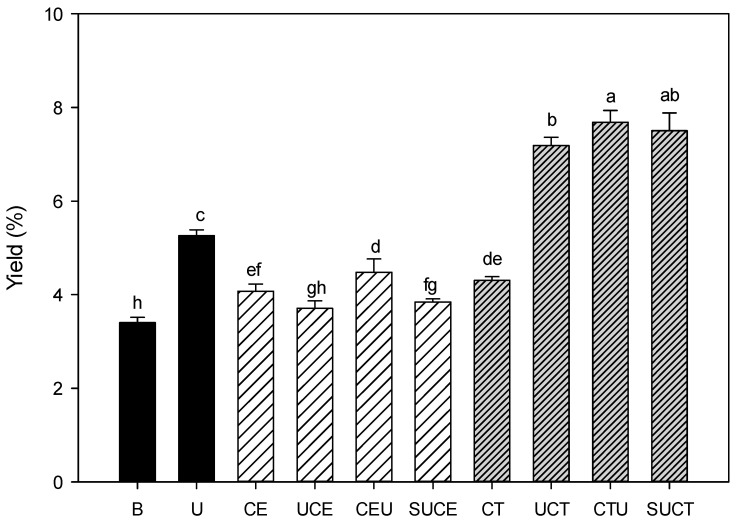
Effect of various extraction methods on the extraction yield of CGO seed mucilage. Sample codes denote the extraction practices. B: citrate buffer extraction, U: ultrasonic extraction, CE: cellulase extraction, CT: Celluclast extraction, UCE: ultrasonic extraction followed by cellulase extraction, CEU: cellulase extraction followed by ultrasound extraction, SUCE: simultaneous ultrasonic and cellulase extraction, UCT: ultrasonic extraction followed by Celluclast extraction, CTU: Celluclast extraction followed by ultrasound extraction, and SUCT: simultaneous ultrasonic and Celluclast extraction. All data are expressed as the mean with standard deviation bar. Data with different letters (a–h) above the standard deviation bar differ significantly by Duncan’s multiple range test at a confidence interval of 95% (*p* < 0.05).

Effect of various extraction practices on the extraction yield of CGO seed mucilage was shown in Figure 1. The extraction yield of the control sample by citrate buffer extraction was about 3.30%. Enzymatic-assisted extraction (EAE) with cellulase or Celluclast^®^ 1.5 L resulted in a 23–30% increase in extraction efficiency as compared to the control sample, and cellulase and Celluclast^®^ 1.5 L assisted extraction showed comparable extraction yield (*p* > 0.05). It is well established that pectin together with cellulose and hemicellulose are the main polysaccharides of plant cell walls and are arranged in a complicated way together with protein matrix [22,23,24]. The cellulolytic enzyme could hydrolyze the cell wall matrix, resulting in more mucilaginous substrates being released from cell walls [6,8,25]. As compared to EAE, ultrasonic-assisted extraction (UAE) is more effective, as evidenced by a 59% increase in extraction yield due to the cavitation effect [26,27]. However, regarding the ultrasonic-assisted enzymatic extraction practice (UAEE), combination of cellulase and ultrasonic treatments (including UCE, CEU and SUCE practices) did not impart a synergistic effect on extraction yield. It is suspected that some of the pectin macromolecules may be hydrolyzed into smaller fragments by the action of cellulase, and the adjunct degradation of macromolecules by ultrasound may diminish the expected enhanced extraction efficiency. In contrast, combination of Celluclast^®^ 1.5 L and ultrasonic treatments (UCT, CTU, SUCT) enhanced the extraction yield pronouncedly (up to 128% increase) (*p* < 0.05). Celluclast^®^ 1.5 L is a commercial multicatalytic enzyme with high cellulo-, xylanolytic and mannanase activities intended for plant tissue breakdown [28]. It suggested that the hydrolyzed and weakened cell wall of CGO seed hull by multicatalytic enzyme action (Celluclast^®^ 1.5 L) in conjunction with the cavitation effect from ultrasound practice facilitated the erosion of cell wall quite effectively (*p* < 0.05) [16,29].

### 2.2. Scanning Electron Micrograph (SEM)

**Figure 2:**
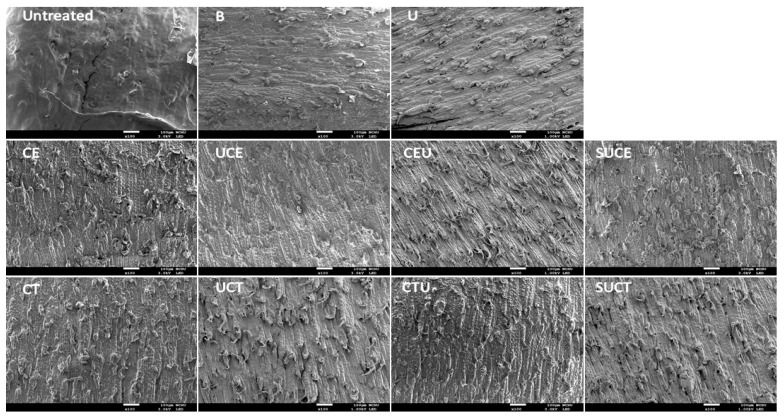
Scanning electron microscopic photographs of the surfaces of CGO seeds after various extraction treatments. Sample codes labeled on the subfigures are the same as described in Figure 1.

The surface microstructures of CGO seed hull after various extraction treatments were examined with scanning electron microscopy (Figure 2). As compared to the untreated seed hull, the surface microstructure of seed hull after citrate buffer extraction was eroded slightly. The extent of surface roughness increased significantly after various UAE, EAE and UAEE practices due to the hydrolysis of the cell wall substrate by enzymatic treatments and/or erosion of cell wall structure by the intense energy of ultrasonic cavitation [30,31]. These results implied that all EAE, UAE and UAEE extraction practices applied effectively enhanced the release of cell wall materials from seed hull and increased extraction efficiency, particularly for UAEE with Celluclast^®^ 1.5 L, which is consistent with the results of extraction yield.

### 2.3. Structural Properties

#### 2.3.1. Proximate Chemical Compositions

Chemical compositions of CGO seed mucilage from various extraction practices were presented in Table 1. The ash and protein contents of CGO seed mucilage varied from about 5.45–7.88% and 0.43–2.90% on dry basis, respectively, implying reasonably high purity of polysaccharide extraction. Mucilage obtained from EAE showed comparable ash content as compared to the control sample (*p* > 0.05). However, extraction practices involved with ultrasound treatment (including UAE and UAEE) generally resulted in mucilage with higher ash content, particularly for UAEE with Celluclast^®^ 1.5 L. This is possibly attributed to the more effective migration of minerals from cell walls by cavitation effect [32]. In addition, mucilage obtained from EAE and UAEE with Celluclast^®^ 1.5 L generally contained higher proteins, implying protein coprecipitation may occur. In studies of EAE of butternut and apple pectin, protein coprecipitation possibly with the arabinose (Ara) and galactose (Gal) residues of pectin sidechains was also reported [25,28,33]. In contrast, mucilage obtained from EAE and UAEE with cellulase generally showed lower protein content, possibly related to the adjunct loss by degradation of some of the pectin macromolecules by the action of cellulase [22,23,24].

#### 2.3.2. Monosaccharide Compositions

As shown in Table 2, the monosaccharide compositions of CGO seed mucilage varied with extraction practices, whereas the major monosaccharide was galacturonic acid (GalA, 48.27–83.36 molar%), implying the CGO seed mucilage is a pectin-like polysaccharide. As compared to citrate buffer extraction, various UAE, EAE and UAEE practices generally reduced GalA and Gal, but increased glucose (Glc), mannose (Man), arabinose (Ara) and rhamnose (Rha) contents, possibly due to the erosion of cell wall matrix resulting in the release of some cellulose fraction or cell wall oligosaccharides and coprecipitated with the mucilage [34]. Generally speaking, the monosaccharide composition is least affected by EAE with Celluclast^®^ 1.5 L, though it showed slightly lower GalA and higher Man contents as compared to the control citrate buffer extracted sample, possibly attributed to the multienzymatic activities of Celluclast^®^ 1.5 L [35]. In contrast, EAE with cellulase dramatically drop the GalA content to less than 60 molar% and increase the Glc content (a foreign sugar molecule for pectin) pronouncedly (from about 4.6 to about 40 molar%), which may be considered as an adverse effect in terms of pectin purity. The intervention of ultrasound cavitation effect in EAE practices complicated the situation due to the increased contact area between phases, resulting in possible cleavage of inter- or intralinkages of macromolecules, and may also impact the enzyme activity [8,29].

#### 2.3.3. FT-IR Analysis and Degree of Esterification of Carboxylic Group

**Figure 3:**
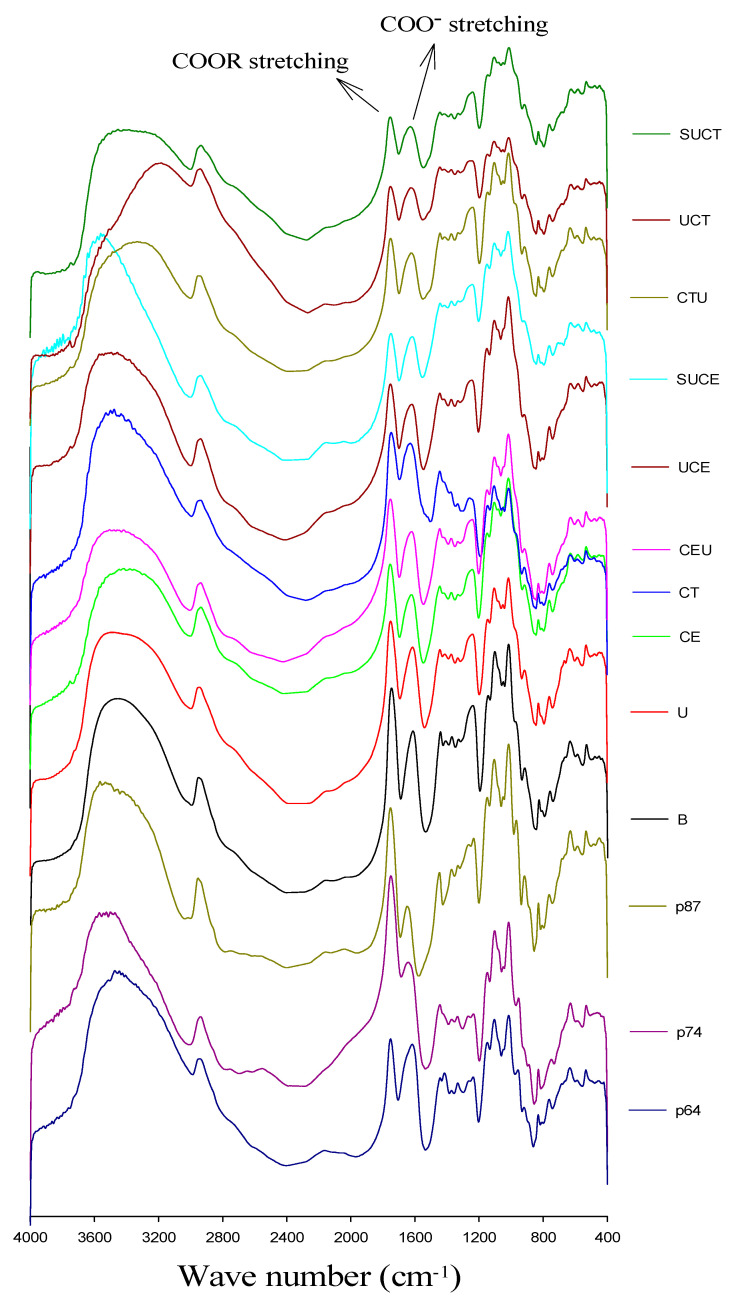
FT-IR spectra for pectin standards and CGO seed mucilage extracted by various extraction methods. P64, P74, and P87 represent pectin standard with a DE of 64, 74 and 87%, respectively. Other sample codes denote the extraction method and are the same as described in Figure 1.

The FT-IR spectra of pectin standards with different degrees of esterification (DE) and CGO seed mucilage from various extraction practices are shown in Figure 3. They all exhibited the typical signals of polysaccharide in the wavenumber range from 4000 to 400 cm^−1^, including the hydrogen bonded O-H at broad band of 3600–3200 cm^−1^, C-H stretching vibration at 2900 cm^−1^. Moreover, a strong peak was observed at 1600–1650 cm^−1^ and 1730–1760 cm^−1^, which were related to the antisymmetric stretching modes of carboxyl ion (COO^−^) and esterified carbonyl groups, respectively [36,37,38]. The degree of esterification (DE) of CGO mucilage was further determined by the ratio of the area of the band around 1749 cm^−1^ (esterified carboxyl) over the sum of the areas of the band around 1749 and 1630 cm^−1^. As shown in Table 3, CGO seed mucilage could be categorized as a high methoxyl pectin-like polysaccharide, as GalA is the major monosaccharide and the DE of carboxyl group is in the range of 72.6–77.7%. As compared to the citrate buffer extracted mucilage, various UAE, EAE and UAEE practices generally reduced the DE of CGO seed mucilage slightly, possibly due to the fact that the citrate buffer concentration (0.5 mM), extraction temperature (53 or 60 °C) and ultrasound conditions applied (1.47 W/mL) are relatively mild. In studies of pectin extraction from tomato pulp and grapefruit peel, researchers found that UAE may cause partial de-esterification [26,27,39]. However, differing results were also reported, possibly related differences in extraction parameters applied, since severe conditions like high temperature or strong acid may facilitate the de-esterification of poly-galacturonic acid [40]. For example, Grassino, et al. [41] showed that UAE operating at 80 °C would lead to de-esterification of pectin, but no significant influence was observed if the UAE was operated at 60 °C. Wikiera, Mika, Starzyńska-Janiszewska and Stodolak [25] found that the catalytic action of purified endo-xylanase resulted in pectin with the highest degree of poly-galacturonic acid methylation (73.4%), exceeding by 17% the DE of pectin obtained with acid-based technique. Yang, Wang, Hu, Xiao and Wu [16] indicated that EAE, UAE and UAEE of pectin from sisal waste resulted in higher DE than acid extraction. Ma, et al. [42] showed that DE of pectin decreased significantly with UAEE as compared to EAE.

#### 2.3.4. Molecular Weight Analysis

Weight average molecular molar mass of CGO seed mucilage obtained from various extraction methods are presented in Table 3. Citrate-buffer-extracted CGO seed mucilage showed much higher molecular weight ($19.03\times 10^{5}$) as compared to pectin from other sources (generally in the range of $10^{4}-10^{5}$), such as orange-peel pectin (5–8 × 10^4^), grapefruit pectin (5–8 × 10^4^) and apple pectin (2–5 × 10^5^) [26,28,43]. In addition to the plant origin, it was possibly attributed to the high GalA purity with low neutral sugar side chains obtained by the relatively mild buffer extraction condition used in this study (0.5 mM at 60 °C). As compared to the citrate buffer extracted mucilage, various UAE, EAE and UAEE practices generally reduced the $\bar{\mathrm{Mw}}$ of CGO seed mucilage, which is expected to impact on the functional properties of CGO seed mucilage. The decrease in $\bar{\mathrm{Mw}}$ is less pronounced for EAE with Celluclast^®^ 1.5 L ($16.31\times 10^{5}$), followed by UAE ($6.69\times 10^{5}$) and UAEE with Celluclast^®^ 1.5 L (5.59–7.65 $\times 10^{5}$). It suggested that the multicatalytic enzyme activity of Celluclast^®^ 1.5 L may cause the hydrolysis of pectin side chain and reduced the $\bar{\mathrm{Mw}}$ slightly. The depolymerization of mucilage by ultrasonic treatment could be ascribed to the cavitation effects initiated in the liquid phase [44]. In contrast, EAE and UAEE with cellulase showed pronounced decrease in $\bar{\mathrm{Mw}}$ $\left(2.06-0.53\times 10^{5}\right)$, implying pronounced hydrolysis by possible presence of residual pectolytic activity in cellulase. In a study of pumpkin pectin extraction with cellulase and hemicellulase, Shkodina, Zeltser, Selivanov and Ignatov [34] also reported that pectin with lower molecular weight was obtained by EAE as compared to the acid extraction ones, possibly due to the partial depolymerization of pectin at the cost of the possible presence of residual pectolytic activity in the cellulase and hemicellulase preparations, and the duration of enzymatic treatment.

### 2.4. Intrinsic Viscosity

Due to the fact that CGO seed mucilage was mainly composed of GalA, the intrinsic viscosity of CGO seed mucilage from various extraction conditions was determined by the method of Chou and Kokini [45], which has been proven to be applicable for ionic polysaccharides [46]. As shown in Table 3, citrate buffer extracted CGO seed mucilage (B) showed much higher intrinsic viscosity (108.64 dL/g) as compared with mucilage from other seed sources such as basil seed mucilage (39.17 dL/g), chia seed mucilage (16.63 dL/g), and flaxseed mucilage (4.46 dL/g) [2,47,48], and pectin from a variety of fruit and vegetable by products (0.75 to 5.9 dL/g) [49]. Intrinsic viscosity is regarded as a measure of the effective hydrodynamic volume of polymers in solutions in the very dilute concentration regime, and is associated with their molecular weight, solubility, chemical compositions (such as uronic acid and protein), and molecular conformation (linear or branched). The much higher intrinsic viscosity of citrate buffer extracted CGO seed mucilage was probably attributed to both the high molecular weight and GalA content, leading to more expanded arrangement of polysaccharides chains by pronounced electric charge repulsion [50]. Whereas, various UAE, EAE and UAEE practices generally reduced the intrinsic viscosity as compared to the citrate-buffer-extracted mucilage. This reduced hydrodynamic volume effect is less pronounced for EAE with Celluclast^®^ 1.5 L (25.72 dL/g), followed by UAE and UAEE with Celluclast^®^ 1.5 L in a decreasing order, and EAE and UAEE with cellulase showed the most pronounced decrease. The decrease in intrinsic viscosity is consistent with the reduction in average molecular weight. In addition, enzymolysis and ultrasonic cavitation of plant cell wall may cause some complex polysaccharide segments and proteins being released and coprecipitated with mucilage, thus modify the charge density, conformation and physicochemical properties of mucilage after various extraction practices [46].

## 3. Materials and Methods

### 3.1. Materials

The fruits of *Citrus grandis* Osbeck (CGO) were purchased from the Farmer Association in Hualien, Taiwan. The seeds of CGO were separated from the pulp, and hot-air-dried at 40 °C until the moisture content was less than 12% (d.b.) and stored in desiccators at room temperature. Cellulase (EC 3.2.1.4, synonyms 1,4-(1,3:1,4)-β-D-Glucan 4-glucanohydrolase with declared activity of about 0.8 units/mg) from *Aspergillus niger* (Sigma Co., St. Louis, MO, USA), Celluclast^®^ 1.5 L (a liquid multicatalytic cellulase with declared activity of 700 EGU/g) (Novozymes, Corp., Beijing, China), citrus pectin standards with known degrees of esterification (67%, 74% and 87%) (Sigma Co., St. Louis, MO, USA) were purchased from the local reagent dealer in Taiwan.

### 3.2. Extraction of Mucilage

Per 8 g of CGO, seeds were mixed with 400 mL of 0.5 mM citrate buffer (pH 5) and then extracted by various extraction treatments as follows. (I) Citrate buffer extraction: the sample solution was shaken in a water bath at 60 °C and 120 rpm for 4 h. (II) Ultrasonic-assisted extraction: the sample solution was sonicated with a 19 mm transducer probe operated under a frequency of 20 kHz and power density of 1.47 W/mL at 53 °C for 1 h (Q700 Sonicator, QSONICA LLC, Newtown, CT, USA) [19,51]. (III) Enzymatic-assisted extraction: The cellulase (a powder cellulase) and Celluclast^®^ 1.5 L (a liquid multicatalytic cellulase) had a declared activity of about 0.8 units/mg and 700 EGU/mL according to the data sheets of the manufacture, respectively. Forty units of cellulase or 35 EGU of Celluclast^®^ 1.5 L per gram of seed was applied for enzymatic-assisted extraction. Five units’ lower enzyme activity for Celluclast^®^ 1.5 L was chosen due to it is a multicatalytic cellulase. The sample solution with cellulolytic enzymes were then shaken in a water bath at 60 °C and 120 rpm for 4 h. After extraction, the sample solution was heated at 95 °C for 15 min to inactive enzyme. (IV) Enzymatic-assisted extraction followed by ultrasonic-assisted extraction: the sample solution was first extracted by the aid of cellulolytic enzymes as described in procedure (III) except for the extraction time was shorten to 3 h, then sonicated for 1 h as described in procedure (II). (V) Ultrasonic-assisted extraction followed by enzymatic-assisted extraction: the sample solution was sonicated for 1 h as described in procedure (II), then extracted by the aid of cellulolytic enzymes as described in procedure (III) except for the extraction time was shortened to 3 h. (VI) Simultaneous extraction by enzymatic and ultrasonic assisted extraction: the sample solution was added 40 U cellulase or 35 EGU Celluclast^®^ 1.5 L per gram of seed, then sonicated for 1 h as described in procedure (II).

Various mucilage extracts were then filtrated through a Whatman^®^ No. 4 filter paper, vacuum concentrated to one eighth of the initial volume using a rotary evaporator (Rika SN-2NW, Eyela, Tokyo, Japan) at 50 °C, then mixed with three volumes of 95% (*w*/*w*) ethanol and kept overnight at 4 °C for mucilage precipitation. The precipitated CGO seed mucilage was centrifuged (8000× *g*, 45 min, 4 °C), hot-air-dried at 40 °C until the moisture content was less than 12% (d.b.), milled (RM100, Retsch GmbH & Co., Haan, Germany), sieved through a 40-mesh sieve, and stored in a desiccator. The sample codes and corresponding extraction conditions are summarized in Table 4.

### 3.3. Extraction Yield

The extraction yield (%) of CGO seed mucilage was calculated by the following formula:

(1) $$\mathrm{Yield}\;(\%)=\frac{\mathrm{Weight}\;\mathrm{of}\;\mathrm{mucilage}\;(\mathrm{db})}{\mathrm{Weight}\;\mathrm{of}\;\mathrm{seed}\;(\mathrm{db})}\;\times \;100$$

### 3.4. Scanning Electron Micrograph (SEM)

The CGO seeds prior and after various extraction treatments were freeze-dried at −50 °C to −60 °C under an absolute pressure lower than 50 Pa. Seed hulls were separated, broken down into pieces, then coated with gold using a coater (JEOL-JEC-1600, Auto Fine Coater, Tokyo, Japan) and examined by scanning electron microscope system (JEOL-JSM6700F, Tokyo, Japan) under 3 kV to view the surface structure of the seed hull.

### 3.5. Proximate Compositions

The moisture content and ash content were determined using AOAC method numbers of 32.1.02 and 4.1.10, respectively [52]. The protein content was approximated by using Bradford protein assay kit (Bio-Rad Laboratories, Inc., Hercules, CA, USA) according to Bradford method [27]. Nitrogen-free extract (N.F.E.) content was estimated by the formula of 100—(ash content + protein content) on dry basis.

### 3.6. Monosaccharide Composition Analysis

Monosaccharide compositions were analyzed according to method of Zeng and Lai [28]. Briefly, per 150 mg of mucilage was hydrolyzed by 10 mL of 2 M TFA at 100 °C for 8 h. To obtain a representative sugar hydrolysate, the recovery of monosaccharides in hydrolysate were first checked. For samples with low recovery, a combination of pectinase and acid hydrolysis was used according to the method of Garna, et al. [29]. After hydrolysis of mucilage, *p*-aminobenzoic ethyl ester derivatization reagent was used to derive the monosaccharides [30]. The derived sample solution was injected into an HPLC system equipped with a Gemini-NX C18 (5 μm) column (4.6 × 250 mm, Phenomenex, Inc., Torrance, CA, USA), and an UV detector was used to determine the absorbance of ABEE-labeled sample at 308 nm. The mobile phase used was 90% of 0.04 M potassium buffer (pH 8.9) and 10% of acetonitrile with a flow rate of 0.8 mL/ min. A series of sugar standards, including glucuronic acid (GlcA), galacturonic acid (GalA), galactose (Gal), mannose (Man), glucose (Glc), arabinose (Ara), xylose (Xyl), fucose (Fuc) and rhamose (Rha) (Sigma Co., St. Louis, MO, USA) were prepared in parallel to construct the standard curves for quantification of the sugar contents.

### 3.7. FT-IR and Degree of Esterification

FT-IR spectroscopy of the CGO seed mucilage was carried out by using a Fourier transform infrared spectrophotometer (Thermo Nicolet Nexus, Thermo Fisher Scientific Inc., Waltham, MA, USA). Per 8 mg of sample was mixed with spectroscopic-grade potassium bromide (KBr) to make a total weight of 0.2 g and pressed into disk [31]. The spectra were recorded at a resolution of 4 cm^−1^ with 64 scans and the absorbance mode from 4000 to 400 cm^−1^. The degree of esterification (DE) of mucilage was determined by the ratio of the area of the band around 1749 cm^−1^ (esterified carboxyl) over the sum of the areas of the band around 1749 and 1630 cm^−1^ according to the methods of Chatjigakis, et al. [32] and Manrique and Lajolo [33]. Citrus pectin standards with known degrees of esterification (67%, 74% and 87%) were prepared in parallel to construct the calibration curve of DE.

### 3.8. Molecular Weight Analysis

Molecular weight analysis of mucilage was determined by using the high-performance size-exclusion chromatography system equipped with a TSK-GEL^®^ G5000PWXL column (TOSOH, BIOSCIENCE, Stuttgart, Germany) as described by Zeng and Lai [34] and Chiang and Lai [35]. The analysis was performed by eluting sample solutions with 50 mM NaNO_3_ solution (containing 0.02% NaN_3_) at a flow rate of 0.5 mL/min. A series of dextran molecular weight standards (5–1400 kDa, Fluka, St. Gallen, Switzerland) were prepared in parallel to construct the molecular weight standard curve. The weight average molar mass was calculated as follows:

(2) $$\bar{\mathrm{Mw}}=\frac{{\Sigma \;\mathrm{Mw}}_{i}\times {\;C}_{i}\;}{{\Sigma \;C}_{i}}$$

where Mw_i_ and C_i_ represent the molar mass and total carbohydrate concentration of ith fraction, respectively.

### 3.9. Intrinsic Viscosity

Various concentrations of CGO seed mucilage in the very dilute solution regime (as checked by specific viscosity <1) were prepared with deionized water and filtered through Whatman No. 4 filter paper. Sample solutions were loaded into a Cannon-Fenske glass capillary viscometer (No. 25) (Cannon Instrument Company, State College, PA, USA) and kept in a thermostatic water bath (Tamson TV4000 visibility bath, Tamson Instruments, Bleiswijk, Netherlands) for an equilibrium time of 15 min at 25 °C. The passage time of sample solutions through the capillary lines were recorded for specific viscosity calculation. Intrinsic viscosity was then determined by using Chou-Kokini’s method [36] from the slope of a regression line of specific viscosity versus solution concentrations according to the following equation:

(3) $$\left[\eta \right]=\underset{c\to 0}{\lim}\;\frac{\eta_{\mathrm{sp}}}{C}$$

where [η] is the intrinsic viscosity (dL/g), η_sp_ is the specific viscosity (dimensionless), and C is the concentration of mucilage solution (g/dL)

### 3.10. Statistical Analysis

All experiments were conducted in triplicated and data were expressed as the means ± standard deviation. Analysis of variance was carried out by using the ANOVA procedures of SPSS software (Version 19.0, IBM Corp., Armonk, NY, USA, 2010), and significant differences between the mean values were determined by Duncan’s multiple range test at a confidence interval of 95%.

## 4. Conclusions

This is the first report about the mucilage from *Citrus grandis* Osbeck seed extracted by ultrasonic or enzymatic-assisted extraction. The results show that the CGO seed from the agricultural/processing byproducts is an excellent new source of high methoxyl pectin with high intrinsic viscosity. The structural and performance characteristics of CGO seed mucilage are strongly linked to the extraction practices. Citrate buffer extracted CGO seed mucilage contains 83.36 molar % GalA and presents quite high intrinsic viscosity (108.64 dL/g) as compared with other pectin sources. The extraction yield of CGO seed mucilage could be effectively increased by extraction procedures involved with ultrasound and cellulolytic enzymes, particularly by UAEE with Celluclast^®^ 1.5 L (about 2.3 times). Though extraction procedures involved ultrasound and cellulolytic enzymes generally show a decrease in GalA contents, weight average molar mass and intrinsic viscosity, EAE with Celluclast^®^ 1.5 L is least affected, followed by UAE and UAEE with Celluclast^®^ 1.5 L. This information would be useful for future application of CGO seed mucilage to leverage the demand features of products. More studies should be carried out regarding CGO seed mucilage application in food systems.

## Figures and Tables

**Table 1:** Effect of various extraction methods on the yield and proximate compositions of CGO seed mucilage.

Treatment	Sample Code ^1^	Moisture ^2^ (%)	Ash ^2^ (%)	Protein ^2^ (%)	N.F.E ^3^ (%)
Citrate Buffer	B	11.44 ± 0.21 a	5.51 ± 0.30 e	0.74 ± 0.02 e	93.75
Ultrasound	U	7.87 ± 0.10 c	7.18 ± 0.07 ab	0.79 ± 0.03 e	92.03
Cellulase	CE	9.27 ± 0.17 b	5.45 ± 0.16 e	0.43 ± 0.01 h	94.12
Cellulase + Ultrasound	UCE	9.26 ± 0.02 b	6.23 ± 0.03 cd	0.63 ± 0.02 f	93.14
	CEU	8.90 ± 0.10 b	6.01 ± 0.09 cd	0.61 ± 0.02 fg	93.38
	SUCE	8.37 ± 0.39 c	5.91 ± 0.04 cde	0.56 ± 0.02 g	93.53
Celluclast	CT	9.13 ± 0.22 b	5.43 ± 0.27 e	2.90 ± 0.05 a	91.67
Celluclast + Ultrasound	UCT	9.46 ± 0.62 b	7.42 ± 0.21 ab	2.12 ± 0.04 c	90.46
	CTU	8.37 ± 0.28 c	6.79 ± 0.13 bc	1.87 ± 0.05 d	91.34
	SUCT	7.16 ± 0.41 d	7.88 ± 0.20 a	2.51 ± 0.08 b	89.61

^1^ Sample codes denote the extraction method and are the same as described in Figure 1. ^2^ Data are expressed as the mean ± standard deviation on dry basis (*n* = 3). Values followed by different letters (a–g) within the same column differ significantly by Duncan’s multiple range test at a confidence interval of 95% (*p* < 0.05). ^3^ NFE denotes nitrogen-free extract and is calculated by (100-ash-protein).

**Table 2:** Effect of various extraction methods on the monosaccharide molar percentage of CGO seed mucilage by acid hydrolysis.

Sugar (molar%)			Sample Code ^1^							
	B	U	CE	UCE	CEU	SUCE	CT	UCT	CTU	SUCT
Acidic	83.49 ± 0.69 a ^2^	67.24 ± 0.61 d	49.24 ± 0.17 i	51.45 ± 0.43 h	54.40 ± 0.22 g	50.78 ± 0.44 h	79.06 ± 0.34 b	63.27 ± 0.38 e	74.07 ± 0.49 c	60.82 ± 1.14 f
GlcA	0.20 ± 0.07 d	1.20 ± 0.20 b	0.97 ± 0.27 c	0.89 ± 0.11 c	0.92 ± 0.01 c	0.88 ± 0.06 c	0.33 ± 0.07 d	1.26 ± 0.06 b	1.56 ± 0.09 a	1.48 ± 0.10 a
GalA	83.72 ± 0.36 a	66.04 ± 0.49 d	48.27 ± 0.44 i	50.56 ± 0.32 h	53.47 ± 0.23 g	49.90 ± 0.38 h	78.72 ± 0.35 b	62.01 ± 0.34 e	72.50 ± 0.41 c	59.34 ± 1.23 f
Neutral	16.51 ± 0.69 h	32.76 ± 0.61 f	50.76 ± 0.17 a	48.55 ± 0.43 b	45.60 ± 0.22 c	49.12 ± 0.44 b	20.94 ± 0.34 h	36.73 ± 0.38 e	25.93 ± 0.49 g	39.18 ± 1.14 d
Gal	8.10 ± 0.58 b	1.59 ± 0.06 c	1.29 ± 0.07 c	1.59 ± 0.11 c	1.44 ± 0.01 c	1.38 ± 0.02 c	11.26 ± 0.40 a	1.60 ± 0.08 c	1.61 ± 0.03 c	1.58 ± 0.05 c
Man	0.37 ± 0.08 e	0.57 ± 0.04 e	1.11 ± 0.08 d	1.16 ± 0.12 d	1.01 ± 0.02 d	1.02 ± 0.10 d	2.05 ± 0.11 c	2.64 ± 0.10 b	2.62 ± 0.09 b	2.98 ± 0.26 a
Glc	4.56 ± 0.02 g	23.37 ± 0.50 e	39.75 ± 0.43 a	35.69 ± 0.78 b	34.41 ± 0.12 c	39.32 ± 0.49 a	3.67 ± 0.27 g	24.27 ± 1.22 e	13.53 ± 0.15 f	26.78 ± 0.05 d
Ara	1.72 ± 2.13 e	3.72 ± 0.13 d	5.74 ± 0.45 b	7.41 ± 0.80 a	6.03 ± 0.05 b	4.83 ± 0.01 c	2.13 ± 0.14 e	4.58 ± 0.67 c	4.75 ± 0.22 c	4.24 ± 0.05 cd
Fuc	0.45 ± 0.03 c	1.51 ± 0.10 a	1.09 ± 0.02 b	1.10 ± 0.02 b	1.13 ± 0.03 b	1.12 ± 0.00 b	0.42 ± 0.07 c	1.71 ± 0.33 a	1.60 ± 0.22 a	1.70 ± 0.27 a
Rha	1.30 ± 0.01 e	2.00 ± 0.10 a	1.79 ± 0.09 bc	1.60 ± 0.05 cd	1.59 ± 0.07 cd	1.55 ± 0.03 cd	1.41 ± 0.26 de	1.92 ± 0.19 a	1.83 ± 0.11 ab	1.89 ± 0.17 a

^1^ Sample codes denote the extraction method and are the same as described in Figure 1. ^2^ Data are expressed as the mean ± standard deviation on dry basis (*n* = 3). Values followed by different letters (a–i) within the same row differ significantly by Duncan’s multiple range test at a confidence interval of 95% (*p* < 0.05).

**Table 3:** Effect of various extraction methods on the intrinsic viscosity and molecular weight and degree of esterification of CGO seed mucilage.

Treatment	Sample Code ^1^	DE (%)	Molecular Weight ^2^ (× 10^5^ Da)	Intrinsic Viscosity ^2^ (dL/g)
Citrate Buffer	B	77.73	19.03 ± 1.40 a ^2^	108.64 ± 2.81 a
Ultrasound	U	74.64	6.69 ± 0.55 d	6.73 ± 0.20 c
Cellulase	CE	72.60	2.06 ± 0.05 e	0.88 ± 0.01 d
Cellulase + Ultrasound	UCE	74.45	0.53 ± 0.03 f	0.72 ± 0.03 d
	CEU	75.82	0.92 ± 0.09 f	0.69 ± 0.01 d
	SUCE	75.99	0.67 ± 0.01 f	1.20 ± 0.01 d
Celluclast	CT	74.21	16.31 ± 0.52 b	25.72 ± 0.33 b
Celluclast + Ultrasound	UCT	75.99	7.65 ± 0.06 c	5.40 ± 0.16 c
	CTU	75.89	5.59 ± 0.29 d	5.62 ± 0.05 c
	SUCT	75.83	7.41 ± 0.05 c	5.60 ± 0.18 c

^1^ Sample codes denote the extraction method and are the same as described in Figure 1. ^2^ Data are expressed as the mean ± standard deviation on dry basis (*n* = 3). Values followed by different letters (a–f) within the same column differ significantly by Duncan’s multiple range test at a confidence interval of 95% (*p* < 0.05).

**Table 4:** Sample codes and conditions of various extraction methods under a solid/solvent ratio of 2 g/100 mL ^1^.

Sample Code	Extraction Method	Condition
B	0.5 mM Citrate buffer (pH 5.0)	4 h, 60 °C, 120 rpm
U	Ultrasonic-assisted extraction	1 h, 53 °C, 1.47 W/mL
CE	Cellulase-assisted extraction	4 h, 60 °C, 120 rpm, 40 U/g seed
UCE	Ultrasonic extraction followed by cellulase extraction	1 h ultrasonic + 3 h cellulase assisted extraction
CEU	Cellulase extraction followed by ultrasonic extraction	3 h cellulase + 1 h ultrasonic assisted extraction
SUCE	Simultaneous extraction by ultrasound and cellulase	1 h of Ultrasonic + Cellulase simultaneously
CT	Celluclast-assisted extraction	4 h, 60 °C, 120 rpm, 35 EGU/g seed
UCT	Ultrasonic extraction followed by Celluclast extraction	1 h ultrasonic + 3 h Celluclast assisted extraction
CTU	Celluclast extraction followed by ultrasonic extraction	3 h Celluclast + 1 h ultrasonic assisted extraction
SUCT	Simultaneous extraction by ultrasound and Celluclast	1 h of Ultrasonic + Celluclast simultaneously

^1^ per 8 g of CGO seeds were mixed with 400 mL of 0.5 mM citrate buffer (pH 5.0).

## Data Availability

The data presented in this study are available on request from the corresponding author. The data are not publicly available due to ethical restriction and the intellectual property issue.

## Sample Availability

The samples are not publicly available due to the funding limitation.

## References

[1] Soukoulis C., Gaiani C., Hoffmann L. (2018). Plant seed mucilage as emerging biopolymer in food industry applications. Curr. Opin. Food Sci..

[2] Qian K., Cui S., Wu Y., Goff H. (2012). Flaxseed gum from flaxseed hulls: Extraction, fractionation, and characterization. Food Hydrocoll..

[3] Zhang J., Jia S., Liu Y., Wu S., Ran J. (2011). Optimization of enzyme-assisted extraction of the Lycium barbarum polysaccharides using response surface methodology. Carbohydr. Polym..

[4] Li B., Smith B., Hossain M.M. (2006). Extraction of phenolics from citrus peels: II. Enzyme-assisted extraction method. Sep. Purif. Technol..

[5] Nadar S.S., Rao P., Rathod V.K. (2018). Enzyme assisted extraction of biomolecules as an approach to novel extraction technology: A review. Food Res. Int..

[6] Rostami H., Gharibzahedi S.M.T. (2017). Cellulase-assisted extraction of polysaccharides from Malva sylvestris: Process optimization and potential functionalities. Int. J. Biol. Macromol..

[7] Yin X., You Q., Jiang Z. (2011). Optimization of enzyme assisted extraction of polysaccharides from *Tricholoma matsutake* by response surface methodology. Carbohydr. Polym..

[8] Marić M., Grassino A.N., Zhu Z., Barba F.J., Brnčić M., Brnčić S.R. (2018). An overview of the traditional and innovative approaches for pectin extraction from plant food wastes and by-products: Ultrasound-, microwaves-, and enzyme-assisted extraction. Trends Food Sci. Technol..

[9] Yuliarti O., Matia-Merino L., Goh K.K., Mawson J.A., Brennan C.S. (2011). Effect of Celluclast 1.5 L on the physicochemical characterization of gold kiwifruit pectin. Int. J. Mol. Sci..

[10] Zhao X., Qiao L., Wu A.-M. (2017). Effective extraction of Arabidopsis adherent seed mucilage by ultrasonic treatment. Sci. Rep..

[11] Aguiló-Aguayo I., Walton J., Viñas I., Tiwari B.K. (2017). Ultrasound assisted extraction of polysaccharides from mushroom by-products. LWT-Food Sci. Technol..

[12] Chen C., You L.-J., Abbasi A.M., Fu X., Liu R.H. (2015). Optimization for ultrasound extraction of polysaccharides from mulberry fruits with antioxidant and hyperglycemic activity in vitro. Carbohydr. Polym..

[13] Chua S.C., Tan C.P., Mirhosseini H., Lai O.M., Long K., Baharin B.S. (2009). Optimization of ultrasound extraction condition of phospholipids from palm-pressed fiber. J. Food Eng..

[14] Toma M., Vinatoru M., Paniwnyk L., Mason T. (2001). Investigation of the effects of ultrasound on vegetal tissues during solvent extraction. Ultrason. Sonochem..

[15] Ponmurugan K., Al-Dhabi N.A., Maran J.P., Karthikeyan K., Moothy I.G., Sivarajasekar N., Manoj J.J.B. (2017). Ultrasound assisted pectic polysaccharide extraction and its characterization from waste heads of Helianthus annus. Carbohydr. Polym..

[16] Yang Y., Wang Z., Hu D., Xiao K., Wu J.-Y. (2018). Efficient extraction of pectin from sisal waste by combined enzymatic and ultrasonic process. Food Hydrocoll..

[17] Wang L., Li T., Liu F., Liu D., Xu Y., Yang Y., Zhao Y., Wei H. (2019). Ultrasonic-assisted enzymatic extraction and characterization of polysaccharides from dandelion (*Taraxacum officinale*) leaves. Int. J. Biol. Macromol..

[18] Chen S., Chen H., Tian J., Wang J., Wang Y., Xing L. (2014). Enzymolysis-ultrasonic assisted extraction, chemical characteristics and bioactivities of polysaccharides from corn silk. Carbohydr. Polym..

[19] Wu H., Zhu J., Diao W., Wang C. (2014). Ultrasound-assisted enzymatic extraction and antioxidant activity of polysaccharides from pumpkin (*Cucurbita moschata*). Carbohydr. Polym..

[20] Wang H.-L., Chen W.-Y., Tsai P.-J., Lin C.-Y., Hsu Y.-T., Chen L.-F., Wu W.-Z., Wang W.-C., Yang W.-J., Chang C.-L. (2016). Isolation of acidic mucilage from the outer seed coat of shaddock (*Citrus grandis* Osbeck) and evaluation of its functional properties. Am. J. Plant Sci..

[21] Wang H.L., Tu C.W., Wu W.Z., Lin C.Y., Chen S.Y., Yang W.J., Chang J.C., Lu M.K., Liao W.T. (2018). Isolation a Homogalacturonan from the Outer Seed Coat of Shaddock (*Citrus grandis Osbeck*). Nat. Prod. Commun..

[22] Oechslin R., Lutz M.V., Amadò R. (2003). Pectic substances isolated from apple cellulosic residue: Structural characterisation of a new type of rhamnogalacturonan I. Carbohydr. Polym..

[23] Zykwinska A.W., Ralet M.-C.J., Garnier C.D., Thibault J.-F.J. (2005). Evidence for in vitro binding of pectin side chains to cellulose. Plant Physiol..

[24] Panouillé M., Thibault J.-F., Bonnin E. (2006). Cellulase and protease preparations can extract pectins from various plant byproducts. J. Agric. Food Chem..

[25] Wikiera A., Mika M., Starzyńska-Janiszewska A., Stodolak B. (2016). Endo-xylanase and endo-cellulase-assisted extraction of pectin from apple pomace. Carbohydr. Polym..

[26] Bagherian H., Ashtiani F.Z., Fouladitajar A., Mohtashamy M. (2011). Comparisons between conventional, microwave-and ultrasound-assisted methods for extraction of pectin from grapefruit. Chem. Eng. Process. Process Intensif..

[27] Wang W., Ma X., Xu Y., Cao Y., Jiang Z., Ding T., Ye X., Liu D. (2015). Ultrasound-assisted heating extraction of pectin from grapefruit peel: Optimization and comparison with the conventional method. Food Chem..

[28] Wikiera A., Mika M., Starzyńska-Janiszewska A., Stodolak B. (2015). Application of Celluclast 1.5 L in apple pectin extraction. Carbohydr. Polym..

[29] Capelo J., Maduro C., Vilhena C. (2005). Discussion of parameters associated with the ultrasonic solid–liquid extraction for elemental analysis (total content) by electrothermal atomic absorption spectrometry. An overview. Ultrason. Sonochem..

[30] Ying Z., Han X., Li J. (2011). Ultrasound-assisted extraction of polysaccharides from mulberry leaves. Food Chem..

[31] Zhu Y., Li Q., Mao G., Zou Y., Feng W., Zheng D., Wang W., Zhou L., Zhang T., Yang J. (2014). Optimization of enzyme-assisted extraction and characterization of polysaccharides from Hericium erinaceus. Carbohydr. Polym..

[32] Abid M., Jabbar S., Wu T., Hashim M.M., Hu B., Lei S., Zeng X. (2014). Sonication enhances polyphenolic compounds, sugars, carotenoids and mineral elements of apple juice. Ultrasonics Sonochem..

[33] Fissore E.N., Ponce N.M., Wider E.A., Stortz C.A., Gerschenson L.N., Rojas A.M. (2009). Commercial cell wall hydrolytic enzymes for producing pectin-enriched products from butternut (*Cucurbita moschata*, Duchesne ex Poiret). J. Food Eng..

[34] Shkodina O.G., Zeltser O.A., Selivanov N.Y., Ignatov V.V. (1998). Enzymic extraction of pectin preparations from pumpkin. Food Hydrocoll..

[35] Shallom D., Shoham Y. (2003). Microbial hemicellulases. Curr. Opin. Microbiol..

[36] Chatjigakis A., Pappas C., Proxenia N., Kalantzi O., Rodis P., Polissiou M. (1998). FT-IR spectroscopic determination of the degree of esterification of cell wall pectins from stored peaches and correlation to textural changes. Carbohydr. Polym..

[37] Manrique G.D., Lajolo F.M. (2002). FT-IR spectroscopy as a tool for measuring degree of methyl esterification in pectins isolated from ripening papaya fruit. Postharvest Biol. Technol..

[38] Singthong J., Cui S.W., Ningsanond S., Goff H.D. (2004). Structural characterization, degree of esterification and some gelling properties of Krueo Ma Noy (*Cissampelos pareira*) pectin. Carbohydr. Polym..

[39] Anese M., Mirolo G., Beraldo P., Lippe G. (2013). Effect of ultrasound treatments of tomato pulp on microstructure and lycopene in vitro bioaccessibility. Food Chem..

[40] Koubala B., Mbome L., Kansci G., Mbiapo F.T., Crepeau M.-J., Thibault J.-F., Ralet M.-C. (2008). Physicochemical properties of pectins from ambarella peels (*Spondias cytherea*) obtained using different extraction conditions. Food Chem..

[41] Grassino A.N., Brnčić M., Vikić-Topić D., Roca S., Dent M., Brnčić S.R. (2016). Ultrasound assisted extraction and characterization of pectin from tomato waste. Food Chem..

[42] Ma X., Wang W., Wang D., Ding T., Ye X., Liu D. (2016). Degradation kinetics and structural characteristics of pectin under simultaneous sonochemical-enzymatic functions. Carbohydr. Polym..

[43] Kratchanova M., Pavlova E., Panchev I. (2004). The effect of microwave heating of fresh orange peels on the fruit tissue and quality of extracted pectin. Carbohydr. Polym..

[44] Chen J., Chen L., Lin S., Liu C., Cheung P.C. (2015). Preparation and structural characterization of a partially depolymerized beta-glucan obtained from Poria cocos sclerotium by ultrasonic treatment. Food Hydrocoll..

[45] Chou T.D., Kokini J.L. (1987). Rheological properties and conformation of tomato paste pectins, citrus and apple pectins. J. Food Sci..

[46] Chiang C.-F., Lai L.-S. (2019). Effect of enzyme-assisted extraction on the physicochemical properties of mucilage from the fronds of *Asplenium australasicum* (J. Sm.) Hook. Int. J. Biol. Macromol..

[47] Naji-Tabasi S., Razavi S.M.A., Mohebbi M., Malaekeh-Nikouei B. (2016). New studies on basil (*Ocimum bacilicum* L.) seed gum: Part I—Fractionation, physicochemical and surface activity characterization. Food Hydrocoll..

[48] Timilsena Y.P., Adhikari R., Kasapis S., Adhikari B. (2015). Rheological and microstructural properties of the chia seed polysaccharide. Int. J. Biol. Macromol..

[49] Fishman M.L., Gillespie D.T., Sondney S.M., El-Atawy Y.S. (1991). Intrinsic viscosity and molecular weight of pectin components. Carbohydr. Res..

[50] Haug A., Smidsrod O.J.A.C.S. (1962). Determination of intrinsic viscosity of alginates. Acta Chem. Scand..

[51] Hsu J.-F. (2017). Studies on the Physicochemical Properties of Mucilage from the Seed of *Citrus grandis* Osbeck with Ultrasound and Enzymatic Assisted Extraction. Master’s Thesis.

[52] AOAC (2000). Official Methods of Analysis.

